# The Superfast Human Extraocular Myosin Is Kinetically Distinct from the Fast Skeletal IIa, IIb, and IId Isoforms* [Fn FN2]

**DOI:** 10.1074/jbc.M113.488130

**Published:** 2013-08-01

**Authors:** Marieke J. Bloemink, John C. Deacon, Daniel I. Resnicow, Leslie A. Leinwand, Michael A. Geeves

**Affiliations:** From the ‡ School of Biosciences, University of Kent, Canterbury, CT2 7NJ, United Kingdom and; the § Department of Molecular, Cellular & Developmental Biology, University of Colorado, Boulder, Colorado 80309

**Keywords:** Actin, Fluorescence, Homology Modeling, Kinetics, Muscle, Myosin, Protein Structure-Function, Sequence Alignment

## Abstract

Humans express five distinct myosin isoforms in the sarcomeres of adult striated muscle (fast IIa, IId, the slow/cardiac isoform I/β, the cardiac specific isoform α, and the specialized extraocular muscle isoform). An additional isoform, IIb, is present in the genome but is not normally expressed in healthy human muscles. Muscle fibers expressing each isoform have distinct characteristics including shortening velocity. Defining the properties of the isoforms in detail has been limited by the availability of pure samples of the individual proteins. Here we study purified recombinant human myosin motor domains expressed in mouse C_2_C_12_ muscle cells. The results of kinetic analysis show that among the closely related adult skeletal isoforms, the affinity of ADP for actin·myosin (*K_AD_*) is the characteristic that most readily distinguishes the isoforms. The three fast muscle myosins have *K_AD_* values of 118, 80, and 55 μm for IId, IIa, and IIb, respectively, which follows the speed in motility assays from fastest to slowest. Extraocular muscle is unusually fast with a far weaker *K_AD_* = 352 μm. Sequence comparisons and homology modeling of the structures identify a few key areas of sequence that may define the differences between the isoforms, including a region of the upper 50-kDa domain important in signaling between the nucleotide pocket and the actin-binding site.

## Teaser

**Background:** Characterization of individual muscle myosin isoforms has been limited by the availability of pure samples of isoforms.

**Results:** Human myosin isoforms expressed in mouse cell line all have different ADP affinities.

**Conclusion:** The superfast extraocular myosin is kinetically distinct from the fast skeletal IIa, IId, and IIb isoforms.

**Significance:** This is the first time adult human sarcomeric myosins have been isolated and characterized by transient kinetics.

## Introduction

Human striated skeletal muscles are composed of fused multinucleated cells known as muscle fibers. Adult striated muscle fibers are classified as either slow/type I, fast/type II, or mixed, based on the type of metabolism (aerobic *versus* anaerobic), the maximum shortening velocity, and the contractile protein isoforms expressed (1, 2). Combinations of different fiber types cooperate to produce contractile activity tuned to the functional demands on the muscle. For example, muscles requiring rapid, short term contractile activity, such as the *extensor digitorum longus*, are composed almost entirely of fast/type II fibers, whereas slow postural muscles, such as the *soleus*, contain a large number of slow/type I fibers. Myosin is the motor protein that, together with actin, is responsible for the generation of force and movement by the muscle fiber. The isoform of myosin expressed in a muscle fiber therefore plays a central role in determining the contractile properties of muscle fibers. Specifically, the ATPase activity and maximum shortening velocity of a fiber are highly correlated with myosin isoform composition (3–5). Skeletal muscle myosin consists of two heavy chains (MyHC)^6^ and two pairs of light chains: the regulatory light chains (RLC) and essential light chains (ELC). The C termini of the myosin heavy chains dimerize as a coiled coil (“myosin tail”), whereas the N termini form the two myosin “heads” or “motor domains.” A short neck joins the C-terminal tail of each MyHC to the motor domain, and each neck is stabilized by a pair of light chains (one ELC and one RLC). The motor domain of the MyHC imparts the primary contractile character to the muscle fiber, whereas the ELC and RLC play modulating roles.

Adult human skeletal muscles express four distinct MyHCs, each encoded by a separate gene (6). The expression pattern is species-specific and is also regulated temporally and spatially. Slow/type I fibers predominantly express MyHC-I (known as MyHC-β when expressed in heart muscle). Fast/type II fibers in adult skeletal muscles express a mixture of the MyHC isoforms IIa and IId and, in a few specific muscles, the extraocular isoform (EO). MyHC-EO is thought to be a very fast isoform because of its expression only in uniquely rapid twitch fibers in the extraocular muscles and laryngeal muscles (7–9). As far as we are aware, EO-MyHC has only been found in fibers expressing a mixture of myosin isoforms, and therefore no studies have been done using fibers containing only MyHC-EO (10). A fifth isoform MyHC-IIb, which is the predominant myosin in the fast skeletal fibers of rodents, is not expressed in healthy human muscles; however, the gene is present and encodes a functional motor (11), and its RNA is detectable in the muscles of patients with Duchenne muscular dystrophy (12).

Among the MyHC isoforms most abundantly expressed in mammalian adult fast skeletal muscle (IIa, IId, and IIb), sequence identity is very high, between 91 and 95%. The specialized MyHC-EO shares only 81–82% identity with IIa, IIb, and IId, whereas the MyHC-β is 81 and 71% identical to the fast muscle isoforms and MyHC-EO, respectively (13). These differences are relatively small, holding out the prospect that it may be possible to correlate the differences in properties of the MyHC isoforms with specific changes in sequence. This does, however, require access to preparations of pure MyHC isoforms.

In the adult skeletal muscle of mammals, ∼80% of individual muscle fibers express a single myosin isoform. Thus it has been possible to define the contractile characteristics of muscle fibers expressing a homogeneous MyHC (14). Studies using single muscle fibers have shown that the MyHC present does define the maximum velocity of shortening of both fibers and *in vitro* motility assays (5, 14). However, detailed biochemical kinetics of a homogeneous myosin isoform has been limited by the very small quantities of myosin that can be prepared from individually characterized muscle fibers (15). Until relatively recently, it was not possible to either separate MyHCs from mixtures or express individual recombinant conventional striated muscle myosin. This restriction resulted in the fact that we have limited information about the biochemical properties of individual mammalian striated muscle myosins and even less information about most human muscle isoforms. Although there is some information about the kinetic properties of cardiac and fast skeletal myosins, there is no kinetic information about the human EO and IIb proteins apart from our recent steady-state studies (11).

**SCHEME 1.**
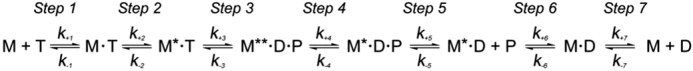
**The interaction of S1 with ATP and ADP.** S1, ATP, and ADP are represented as *M*, *T*, and *D*, respectively. *Asterisks* indicate the different levels of tryptophan fluorescence and represent the different conformational states of the myosin.

**SCHEME. 2:**
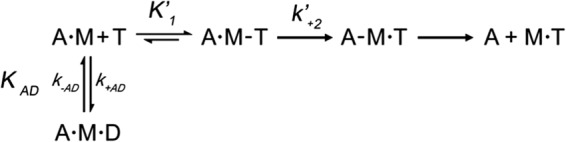
**The interaction of S1 with actin, ATP and ADP.** S1, actin, ATP, and ADP are represented as *M*, *A*, *T*, and *D*, respectively. Dashed (-) interactions represent a weakly bound complex, and dotted (·) interactions represent strongly bound states. Cross-bridge detachment from the rigor state (A·M) involves the complex binding ATP, governed by the association constant, *K*′_1_, followed by the rate-limiting isomerization, *k*′_+2_, after which actin-myosin affinity becomes weak, and the complex separates rapidly (22, 23).

Winkelmann and co-workers (16, 17) demonstrated that a muscle cell system was required for proper folding of striated muscle myosins and used mouse C_2_C_12_ cells to express chicken embryonic myosin. More recently, we developed this mammalian muscle cell expression system further to produce active human MyHC motor domains with associated light chains (11, 18). We refer to these constructs as MyHC-S1, analogous to subfragment 1 prepared from muscle tissue. In our previous work using recombinant S1 proteins produced with this expression system, the ATPase and *in vitro* motility properties of the individual human skeletal muscle MyHC isoforms were shown to be distinct (11). In this study, we use the same constructs to complete a more thorough biochemical kinetic characterization of the MyHCs and correlate the properties of each MyHC with the differences in motor domain sequence. Transient kinetics measurements define the rate and equilibrium of various steps in the cross-bridge cycle (Schemes 1 and 2). We have used the three recombinant human adult fast skeletal MyHC isoforms, IIa, IId, and IIb, as well as the specialized MyHC-EO. We contrast the new data collected here with our previous data on the human cardiac α and β (skeletal isoform I) isoforms (18). The results show that EO has very fast kinetics and is quite distinct from the other three fast muscle isoforms. All four isoforms are distinct from the slow muscle MyHC-I (β-cardiac isoform), whereas the α-cardiac isoform shares many kinetic properties with the fast muscle isoforms, although it has a distinct sequence. Of note also is that the IIb isoform is a functional protein but does not appear to be similar to IIb from small mammals despite high sequence similarity.

## EXPERIMENTAL PROCEDURES

### 

#### 

##### Proteins

Human skeletal muscle MyHC-S1 proteins were expressed and purified as described recently (11). Briefly, active recombinant human skeletal muscle MyHC-S1 (residues Met-1 to Pro-843) was expressed in the C_2_C_12_ myoblast cell line (transformed murine muscle cell precursors) following differentiation of C_2_C_12_ cells in culture into functional myotubes. Replication incompetent recombinant adenoviruses were produced using the AdEasy system (Qbiogene) containing expression cassettes encoding S1 of the human skeletal myosins, with a C-terminally fused enhanced GFP (eGFP) and His_6_ tag, under the transcriptional control of a CMV promoter. These adenoviruses were used to infect C_2_C_12_ myotubes in culture and resulted in overexpression of recombinant myosin proteins. S1-eGFP-His_6_ proteins were purified in three steps. Infected C_2_C_12_ cells were lysed using a Dounce homogenizer in low salt lysis buffer to precipitate endogenous, full-length C_2_C_12_ myosins. Lysates were clarified by centrifugation then subjected to nickel affinity chromatography. Eluates were dialyzed into low salt buffer and then subjected to anion exchange chromatography by FPLC. We isolated proteins from ∼1500 to 3000 cm^2^ of cultured C_2_C_12_ cells per preparation, yielding up to ∼1 mg of purified MyHC per preparation at up to ∼10 μm final concentration.

As reported by Resnicow *et al.* (11), the S1 proteins purified by this method are isolated in complex with the mouse ELC and RLC proteins present in the C_2_C_12_ cells except the MyHC-EO, which has no light chains present. SDS-PAGE from protein preparations were similar to those of Resnicow *et al.* (11), and typical gels are shown in the supplemental materials. These gels show the presence of three LC bands previously identified as a combination of mouse light chains 1, 2, and 3 plus the atrial/fetal light chain. The ratio of ELC to RLC was always close to 1:1 (see supplemental Table 1 for details). Rabbit skeletal muscle actin was prepared according to methods described previously (19) and labeled with pyrene at Cys-374 (20).

##### Transient Kinetics

All kinetic experiments were performed at 20 °C in 20 mm MOPS buffer, pH 7.0, with 100 mm KCl, 5 mm MgCl_2_, and 1 mm azide, unless indicated otherwise. Measurements were performed with a High-Tech Scientific SF-61 DX2 stopped flow system. Pyrene-actin fluorescence was excited at 365 nm, and emission was detected after passing through a KV389-nm cut-off filter (Schott, Mainz, Germany). A Kodak 47B filter was used in combination with the KV389 filter to avoid interference of the eGFP fluorescence with the pyrene fluorescence signal. Tryptophan fluorescence was excited at 295 nm and observed through a WG320 filter. The stated concentrations of reactants are those after mixing in the stopped flow observation cell, unless otherwise specified. Stopped flow data were analyzed using the software provided by TgK Scientific (Kinetic Studio), as well as with Origin (Microcal). Without actin present, the kinetics of MyHC-S1 with ATP (T) or ADP (D) were interpreted based on the seven-step model described by Bagshaw *et al.* (21), where *k_+i_* and *k*_−_*_i_* are the forward and reverse rate constants, and *K_i_* (= *k_+i_*/*k*_−_*_i_*) represents the equilibrium constant of the *i*th step of the reaction (Scheme 1).

In the presence of actin, the kinetics of the interaction of S1 with ATP or ADP were analyzed based on the model previously developed for myosin (22, 23). As shown in Scheme 2, ATP binds rapidly and reversibly to actin-myosin, followed by a rate-limiting isomerization (*k*′_+2_) of the complex, which leads to rapid dissociation of actin. ADP competes with ATP for the nucleotide-binding site, and its binding to actin-myosin is governed by the dissociation constant, *K_AD_* (= *k*_−_*_AD_*/*k*_+_*_AD_*).

Pyrene-actin fluorescence is quenched in the pyrene-actin-S1 complex. Therefore, an increase in fluorescence intensity can be observed as a result of pyrene-actin dissociation from S1. The dissociation reaction can be induced by the addition of ATP to the pyrene-actin-S1 complex, and resulting fluorescence transients can be analyzed to define the kinetics of the reaction. Equation 1 was derived from the interaction of actin·S1 with ATP (Scheme 2) and was used to analyze ATP-induced actin·S1 dissociation to determine the constants, *K*′_1_*k*′_+2_, *k*′_+2_, and 1/*K*′_1_.

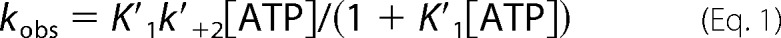

Equation 2 was derived from the interaction of actin·S1 with ATP and ADP (Scheme 2) and assumes that ADP is in rapid competition with ATP for binding to AM and *K*′_1_[ATP] < 1, such that the equation is linear with respect to [ATP]. The equation was used to analyze the data for ATP induced dissociation of actin from the complex in the presence of ADP.

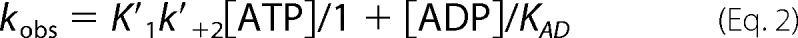
 If the *k*_obs_ in the absence of ADP is *k*_o_, then Equation 2 can be normalized to make comparison of different myosin isoforms easier.

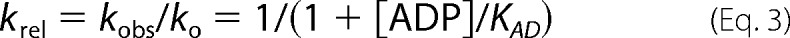
 The two amplitudes observed in the ADP displacement from S1 are proportional to the concentration of the S1 present as free S1 or S1·ADP. The amplitudes depend on the total ADP concentration as defined as follows:

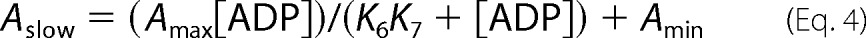


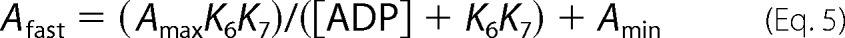


In all cases, the data in the figures refer to individual experimental measurements, whereas Table 1 gives the mean values of the fitted constant for two to three separate measurements using different protein preparations.

##### Sequence Alignments and Homology Modeling

Three-dimensional homology models were generated for the human skeletal myosin isoforms IIa, IIb, IId, and EO using the SWISS-MODEL automatic comparative protein modeling server (24, 25). The primary sequences of the human isoforms were pairwise aligned with the sequence of three scallop myosin head structures as templates (Protein Data Bank codes 1kk8, 1qvi, and 1sr6) using the CLUSTALW alignment protocol. The alignments were submitted to the alignment interface of SWISS-MODEL, and the generated models were validated using WHAT CHECK (26). A sequence identity of 60% (±0.5%) was found when aligning the human skeletal myosin head sequences with class II scallop myosin, allowing us to build well resolved homology models (27). The scallop myosin structures used as templates were chosen because they represent various conformational states of myosin during the cross-bridge cycle: the post-power stroke state containing ADP-BeF_x_ in the nucleotide pocket (Protein Data Bank code 1kk8), the pre-power stroke state containing ADP-VO_4_ (Protein Data Bank code 1qvi), and one scallop myosin structure without a nucleotide in the binding pocket (Protein Data Bank code 1sr6) representing the near rigor state of myosin.

## RESULTS

### 

#### 

##### The Affinity of EO for ATP Is Weaker than IIa, IIb, and IId in the Presence of Actin

**FIGURE 1.**
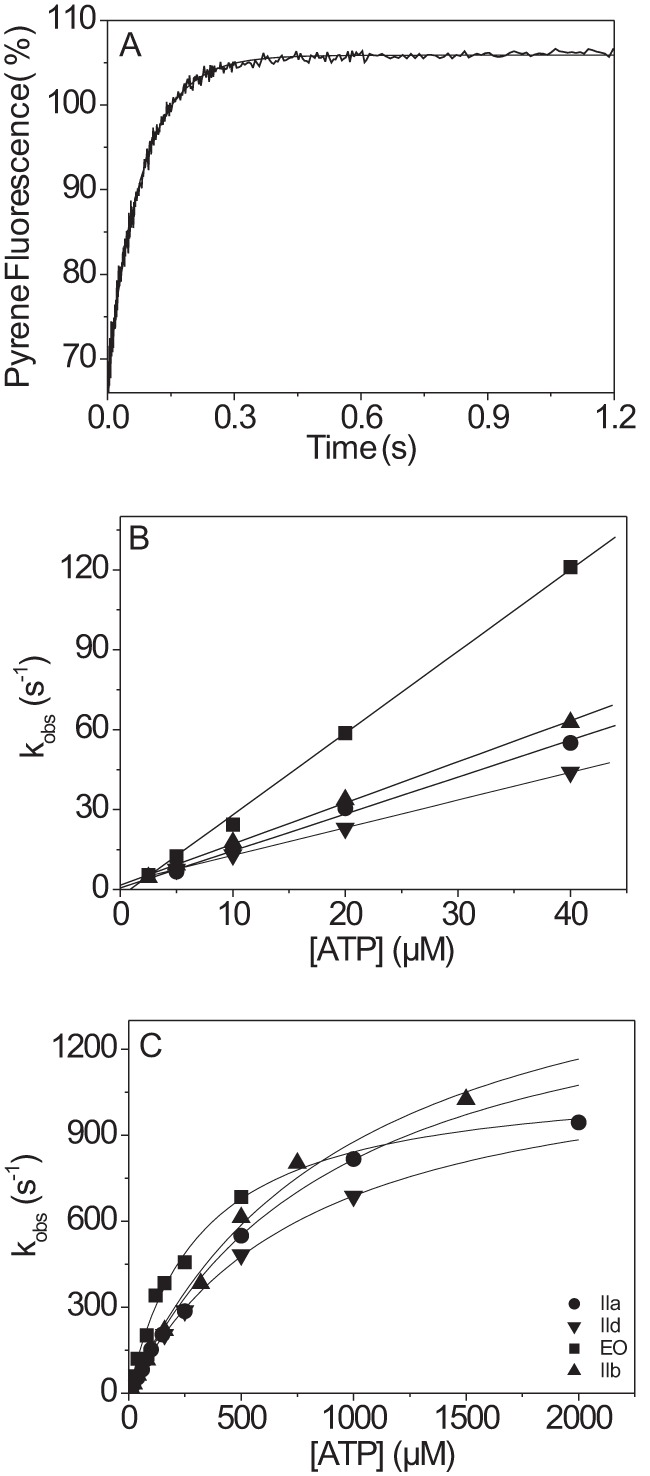
**ATP-induced dissociation of actin·S1.** *A*, example trace of the pyrene fluorescence changes observed on mixing 0.25 μm pyrene-labeled actin·EO-S1 complex with 5 μm ATP at 20 °C. The best fit single exponential is superimposed with *k*_obs_ = 12.4 s^−1^ (and amp = +36%). *B*, at low ATP concentrations, the ATP concentration dependence was described by a straight line with slope *K*′_1_*k*′_+2_. For EO-S1 (■), *K*′_1_*k*′_+2_ = 3.2 μm^−1^ s^−1^ and for IIa (●), IIb (▴), and IId (▾), *K*′_1_*k*′_+2_ = 1.7 μm^−1^ s^−1^. *C*, the dependence of *k*_obs_ *versus* [ATP] fitted to Equation 1 shows the maximum rate of dissociation *k*_max_ = 1000–1200 s^−1^ for the isoforms IIa, IIb, IId, and EO.

ATP binding to actin·S1 was determined for each myosin isoform using rapid mixing in a stopped flow apparatus. The ATP-induced dissociation of actin·S1 complexes was followed by monitoring the fluorescence of a pyrene label covalently attached to Cys-373 of actin. Fig. 1*A* shows an example of the pyrene fluorescence changes observed upon mixing 0.25 μm pyrene-labeled actin·EO-S1 with 5 μm ATP at 20 °C. The signal was fitted using a single exponential equation resulting in *k*_obs_ = 12.4 s^−1^ and amplitude = +36%. Varying the ATP concentration and plotting *k*_obs_ as a function of ATP concentration (range, 2.5–40 μm) results in a linear relationship between *k*_obs_ and allowed the determination of the second order rate constant of ATP binding to actin·S1 (*K*′_1_*k*′_+2_, = 3.5 ± 0.2 μm^−1^ s^−1^ see Fig. 1*B*). Over a wider concentration range, the relation between *k*_obs_ and [ATP] was described by a hyperbola as predicted in Equation 1 (see Fig. 1*C*). Fitting the EO data yielded, a maximum rate constant of ATP induced dissociation *k*′_**+**2_ = 1152 ± 60 s^−1^ and an apparent ATP affinity for AM (1/*K*′_1_ = 318 μm). The same method was used to measure the other human skeletal myosin isoforms (IIa, IIb, and IId), the results are depicted in Fig. 1 (*B* and *C*), and the average values of the fitted constants for at least three different preparations of each isoform are summarized in Table 1. The data show that the skeletal muscle isoforms IIa, IIb, and IId have identical second order rate constants (*K*′_1_*k*′_+2_ = 1.7 μm^−1^ s^−1^), whereas for EO this parameter was 2-fold higher (*K*′_1_*k*′_+2_ = 3.2 μm^−1^ s^−1^). The values for the maximum rate constant of ATP-induced dissociation are similar for the four different isoforms (*k*′_+2_ = 1000–1200 s^−1^). The difference in *K*′_1_*k*′_+2_ between EO and the other three skeletal isoforms is due to an approximate 2-fold decrease in the value of 1/*K*′_1_: 360 μm for EO and 600–760 μm for the other three isoforms.

**TABLE 1:** **Transient kinetic parameters measured for EO, IIa, IIb, and IId myosin S1 isoforms** The values are means ± S.D. based on a minimum of three different protein preparations. All measurements were performed at 20 °C in 100 mm KCl, 20 mm MOPS, 5 mm MgCl_2_ (pH 7).

	EO-S1	IIa-S1	IIb-S1	IId-S1	β-S1	β-S1*^a^*	α-S1*^a^*
**ATP binding to S1**							
*K*_1_*k*_+2_ (μm^−1^ s^−1^)	1.0 ± 0.2*^b^*^,^*^c^*	2.5 ± 0.5*^c^*^,^*^d^*	1.7 ± 0.1*^b–d^*	3.8 ± 0.2*^b^*^,^*^d^*	1.38	1.5 ± 0.1	2.2 ± 0.1
*k*_+2_ or *k*_+3_ +*k*_−3_ (s^−1^)	177 ± 8	141 ± 28	169 ± 2	195 ± 11	179	158 ± 18	168 ± 28

**ADP binding to S1**							
*K*_6_*K*_7_ (μm)	0.7 ± 0.2	1.0 ± 0.3	1.7 ± 0.3	3.0 ± 0.5	2.5	0.5 ± 0.1	2.8 ± 0.7
*k*_+6_ (s^−1^)	0.7 ± 0.1	0.9 ± 0.1	1.7 ± 0.4	1.5 ± 0.2	1.0	0.9 ± 0.1	2.7 ± 0.6

**ATP binding to acto-S1**							
*K*′_1_*k*′_+2_ (μm^−1^ s^−1^)	3.2 ± 0.4	1.7 ± 0.1*^d^*	1.7 ± 0.3*^d^*	1.7 ± 0.6*^d^*	1.35	1.1 ± 0.1	2.5 ± 0.3
*k*′_+2_ (s^−1^)	1152 ± 60	1125 ± 78	1216 ± 283	1023 ± 215	1070	1450 ± 150	1500 ± 167
1/*K*′_1_ (μm)	360	662*^d^*	715*^d^*	602*^d^*	764	1140 ± 65	626 ± 143

**ADP binding to acto-S1**							
*K_AD_* (μm)	352 ± 9	80 ± 15*^e^*^,^*^e^*	54 ± 11*^e^*	118 ± 33*^e^*^,^*^f^*	14	21 ± 5	152 ± 25
*k*_−_*_AD_* (s^−1^)	>1100	>1100	>1200	>1000		93 ± 5	>1200

*^a^* Human α- and β-S1 without the eGFP tag. The data are from Ref. 18.

*^b^ p* < 0.05 determined by Student's *t* test as compared to IIa-S1.

*^c^ p* < 0.05 determined by Student's *t* test as compared to IId-S1.

*^d^ p* < 0.05 determined by Student's *t* test as compared to EO-S1.

*^e^ p* < 0.0001 determined by Student's *t* test as compared to EO-S1.

*^f^ p* < 0.05 determined by Student's *t* test as compared to IIb-S1.

##### The Affinity of Actin·MyHC-EO for ADP (K*_AD_*) Is More than 3-fold Weaker Compared with IIa, IIb, and IId

**FIGURE 2.**
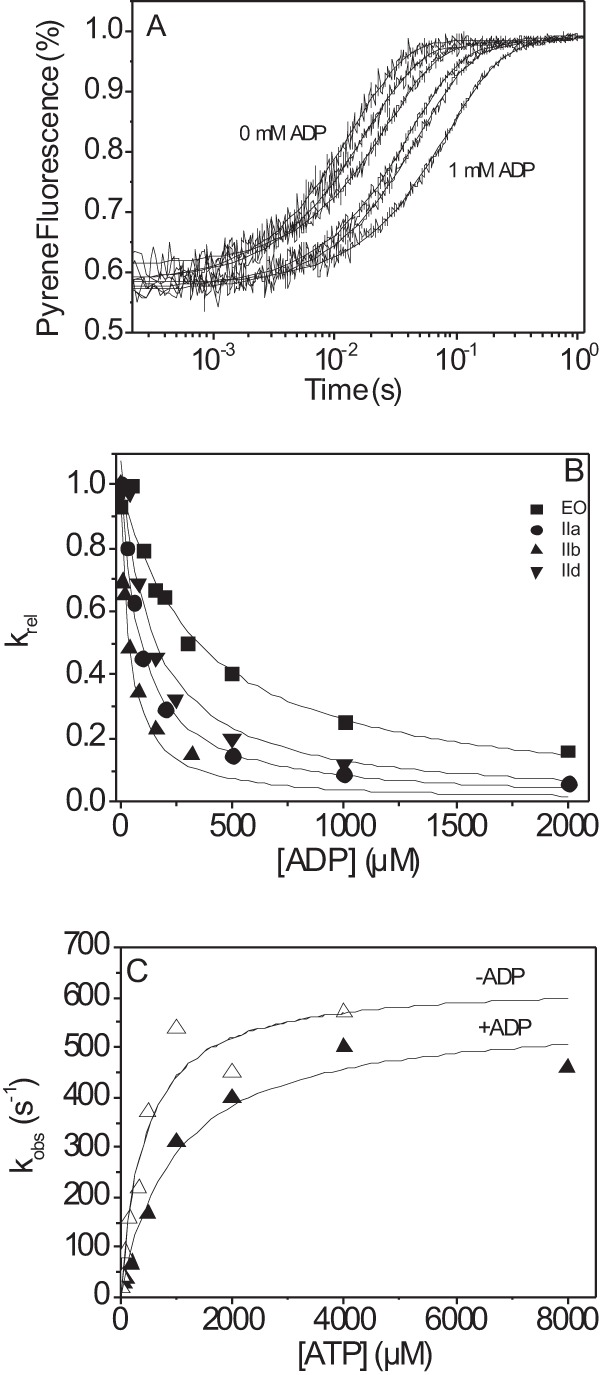
**ATP-induced dissociation of pyrene-actin·S1 in the presence of ADP.** *A*, pyrene fluorescence traces observed on mixing 0.25 μm pyrene-actin·S1-IIa with 50 μm ATP and variable [ADP] at 20 °C. The best fit single exponential yields *k*_obs_ for each [ADP]. *B*, *k*_obs_ as a function of [ADP] for four skeletal myosin S1 isoforms fitted to Equation 2. The ADP affinity *K_AD_* decreases from *K_AD_* = 54 ± 11 μm (IIb, ▴), *K_AD_* = 80 ± 15 μm (IIa, ●), *K_AD_* = 118 ± 33 μm (IId, ▾), to *K_AD_* = 352 ± 9 μm (EO, ■). *C*, ATP-induced dissociation of actin·S1-IIb in the presence (▴) or absence (▵) of 100 μm ADP at 12 °C. A hyperbolic fit at high [ATP] gives similar maximum *k*_obs_ for the dissociation of actin·S1-IIb in the presence or absence of ADP (*k*′_+2_ = 597 ± 40 s^−1^ and 630 ± 55 s^−1^, respectively).

The affinity of ADP (*K_AD_*) for each actin·S1 complex was measured by competition with ATP binding. Pyrene-labeled actin·S1 at a concentration of 0.125 μm was rapidly mixed with 50 μm ATP that had been premixed with increasing concentrations of ADP. Fig. 2*A* shows a representative trace of the pyrene fluorescence measured using the IIa-S1 isoform. The fluorescence traces were fitted using a single exponential equation, and the resulting *k*_obs_ values were plotted against ADP concentration and fitted using a hyperbolic function (Equation 2), resulting in an apparent affinity (*K_AD_*) for ADP (Fig. 2*B*). This measurement was repeated for all S1 isoforms, and the results are summarized in Fig. 2*B* and Table 1. The affinity of actin-S1 for ADP (*K_AD_*) varied from 54 to 118 μm for the three fast muscle isoforms; the EO isoform had a much weaker ADP affinity (*K_AD_* ≈ 350 μm). The ADP binding to actin-S1 (*K_AD_*) is normally considered to be a rapid equilibrium reaction with a diffusion limited rate constant of binding (*k*_+_*_AD_* > 10^7^ m^−1^ s^−1^) (28) and the ADP release rate constant (*k*_−_*_AD_*), estimated from *K_AD_*/*k*_+_*_AD_*, but normally > 1000 s^−1^. Because S1-IIb has the tightest ADP affinity of the four fast skeletal muscle isoforms (IIb > IIa > IId > EO), we tested whether ADP release remained very fast for this isoform. Fig. 2*C* shows the ATP-induced dissociation of actin·S1-IIb in the presence or absence of 50 μm ADP. We used a lower temperature (12 °C) to improve the precision of the maximum value of *k*_obs_ that can be measured. A plot of the *k*_obs_ *versus* [ATP] is shown in Fig. 2*C*, and the maximum value for *k*_obs_ saturates at approximately the same value in the presence and absence of ADP (567 ± 40 and 630 ± 55 s^−1^, respectively). Thus ADP release (*k*_−_*_AD_*) is much faster than the maximum rate constant of ATP binding *k*′_+2_ for actin·S1-IIb·ADP (>630 s^−1^ at 12 °C or >1200 s^−1^ at 20 °C). ADP binding is therefore a rapid equilibrium step and is not rate-limiting for the dissociation of actin·S1 at high ATP concentrations. Because the other isoforms were found to have weaker affinity for ADP compared with IIb, the ADP release step is not rate-limiting for the ATP-induced dissociation of actin·S1 at high ATP concentrations.

##### ATP Binding to EO-S1 Is Slower than to IIa, IIb, and IId

**FIGURE 3.**
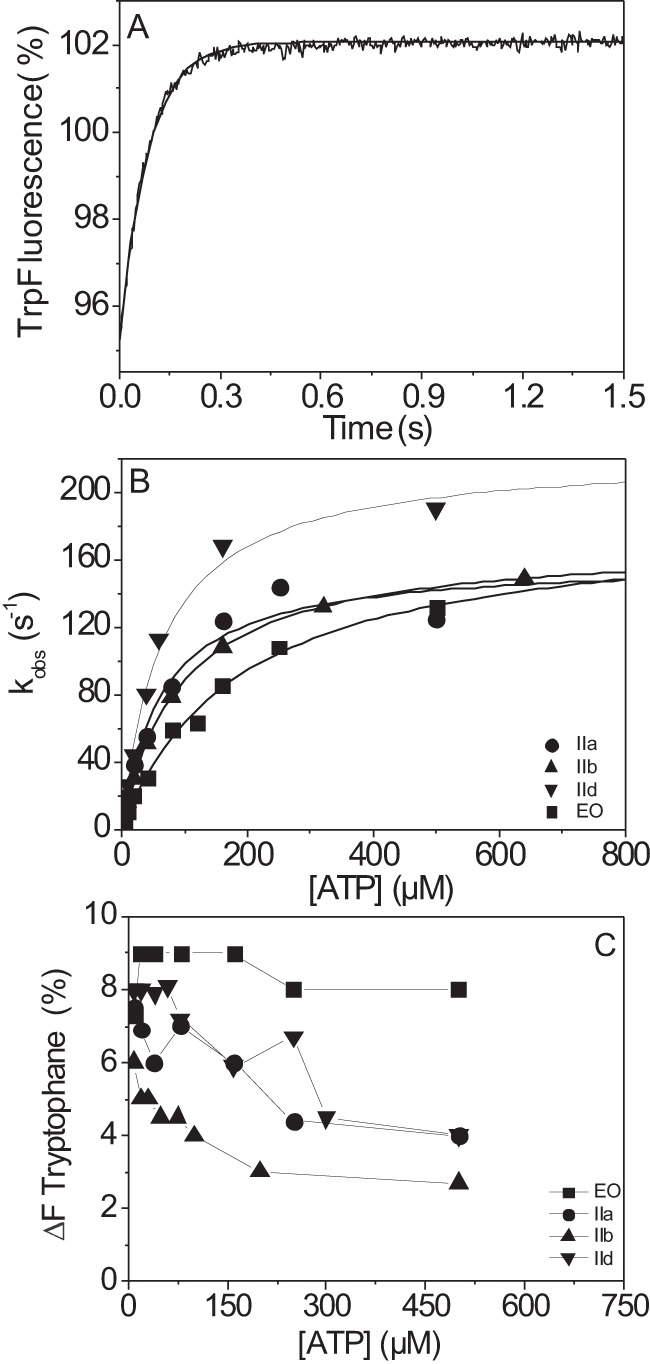
**Rate of association of ATP with fast skeletal MyHC-S1.** *A*, the protein fluorescence changes observed on mixing 0.25 μm S1-IId with 5 μm ATP at 20 °C. The fluorescence signal could be fitted using a single exponential with *k*_obs_ = 12.3 s^−1^ and an amplitude of 6.8%. *B*, dependence of *k*_obs_ on ATP concentration is depicted for skeletal myosin S1 isoforms IIa (●), IIb (▴), IId (▾), and EO (■). At high ATP concentrations, *k*_obs_ saturates at between 140 and 195 s^−1^. *C*, measured amplitudes as a function of ATP concentration.

The binding of ATP or ADP to S1 is characterized by an increase in intrinsic protein fluorescence associated with nucleotide binding and hydrolysis. Fig. 3*A* shows a representative trace of the fluorescence change observed on rapidly mixing 0.25 μm IId-S1 with 5 μm ATP at 20 °C. The fluorescence signal was fitted to a single exponential with *k*_obs_ = 12.3 s^−1^ (amplitude 6.8%). Varying the ATP concentration and plotting *k*_obs_ as a function of [ATP] results in the second order rate constant of ATP-binding IId-S1, *K*_1_*k*_+2_ = 3.8 ± 0.2 μm^−1^ s^−1^ and a maximum rate of ATP binding, *k*_max_ = 195 ± 11 s^−1^ (Fig. 3*B*). The same method was used to measure the other human skeletal myosin isoforms, and the results are shown in Fig. 3*B* and Table 1. In contrast to the data for actin·S1, the second order rate constant for ATP binding to S1 is slowest for EO-S1 and varies from EO < IIb < IIa < IId. The maximum rate of the fluorescence change also shows modest variation from 141 to 195 s^−1^ and follows the order from fastest to slowest: IId > EO > IIb > IIa. The value of *k*_max_ can be assigned to either the maximum rate of ATP binding *k*_+2_ or the hydrolysis step (*k*_+3_ + *k*_−3_), and this will be considered in the discussion.

##### The Affinity of S1 for ADP Is Tighter for EO than for IIa, IIb, and IId

**FIGURE 4.**
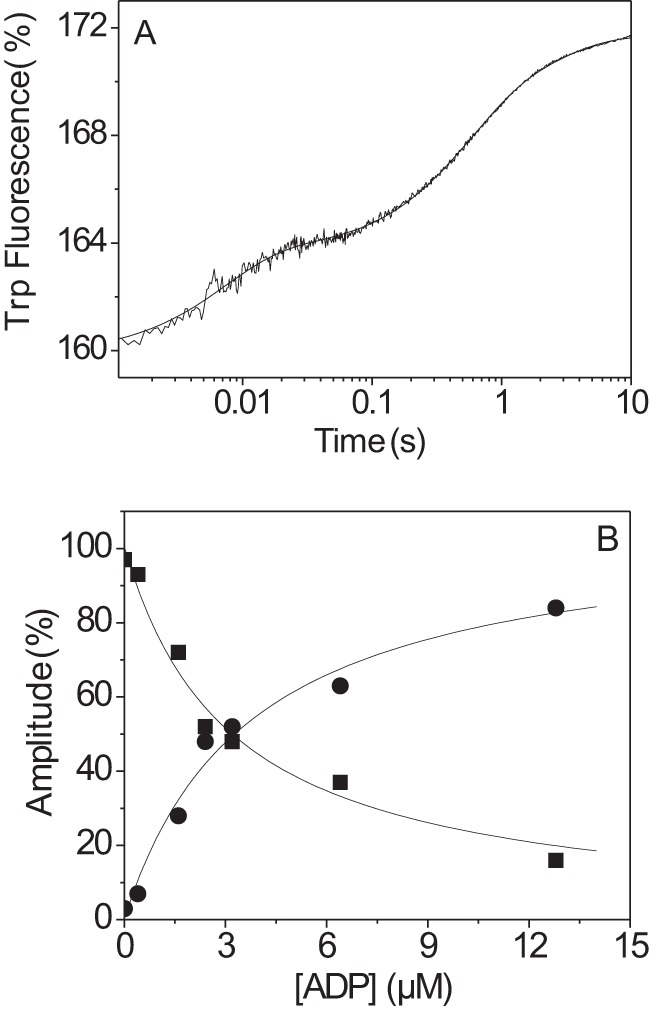
**Displacement of ADP from S1-IId by excess ATP.** *A*, the protein fluorescence changes observed after mixing 0.25 μm S1-IId, preincubated with 3.2 μm ADP with 100 μm ATP. The fluorescence signal was fitted using a double exponential function with a fast phase (*k*_obs_ = 144 s^−1^ and amp = 2.4%) and a slow phase (*k*_obs_ = 1.6 s^−1^ and amp = 4%). *B*, dependence of the amplitudes of the fast and slow phase on [ADP], resulting in *K_D_* = 3.2 ± 0.4 μm for the fast phase (■) and *K_D_* = 3.7 ± 0.4 μm for the slow phase (●).

The ADP affinity of MyHC-S1 (*K_D_* = *K*_6_*K*_7_ of Scheme 1) was measured indirectly by preincubating the protein with various amounts of ADP and then subsequent mixing with excess ATP. Two phases are expected in the tryptophan fluorescence trace: a fast phase, caused by the binding of ATP to S1 that has no ADP bound, and a slow phase, caused by the binding of ATP to S1-ADP, which is limited by the rate of ADP dissociation (or *k*_+6_). This is depicted in Fig. 4*A* for the reaction of 0.25 μm IId-S1, preincubated with 3.2 μm ADP, and then mixed 100 μm ATP. The fluorescence trace was fitted using a double exponential equation with a fast (*k*_obs_ = 144 s^−1^) and a slow rate constant (*k*_obs_ = 1.6 s^−1^) with amplitudes of 2.4 and 4% for the fast and slow phase, respectively. Increasing the concentration of ADP did not alter the *k*_obs_ for either phase but did change the amplitudes of both the fast and the slow phase as depicted in Fig. 4*B*. Fitting the amplitude data using Equations 4 and 5 allowed the affinity of IId-S1 for ADP (*K_D_* = *K*_6_*K*_7_; Scheme 1) to be determined, resulting in *K_D_* = 3.0 ± 0.5 μm. The measurement was repeated for the other three MyHC-S1 constructs and resulted in the *K_D_* values between 0.7 and 3 μm as listed in Table 1. The ADP dissociation rate constants (*k*_−6_) varied over the range 0.7–1.7 s^−1^ and were closely correlated with the measured ADP affinity for each isoform.

##### Influence of the eGFP Tag

The eGFP tag was added on the original protein to aid isolation of the protein and used to attach the protein to surfaces via a GFP antibody for the motility assays. We used that same construct to allow comparison to the data of Resnicow *et al.* (11). The presence of the eGFP tag on the fast skeletal myosin constructs necessitated the use of the Kodak 47B filter for fluorescence measurements on the stopped flow to minimize the interference between the GFP and pyrene signals. To investigate the influence of the eGFP on our experiments, we performed measurements on a MyHC-β-S1-eGFP fusion and compared these values to our previously reported data from a MyHc-β-S1 construct lacking the eGFP tag (18). Comparison of the measured parameters for the β-S1 constructs with and without this eGFP tag identified only one kinetic parameter that was substantially different: the ADP affinity for S1 without actin present; *K_D_* was 5-fold tighter in the absence of the tag (see Table 1). All other measured constants were consistent to within 20% with and without the GFP tag (Table 1). ADP displacement had the smallest fluorescence signal amplitude for the reactions measured, and therefore any influence of eGFP fluorescence on the tryptophan fluorescence transients would be most significant for such data.

##### Sequence Alignments and Homology Models

Sequence alignments were made for the four human MyHC-S1 constructs (IIa, IIb, IId, and EO) together with human cardiac α and β (see supplemental Fig. S2) (18). The three areas known to be hypervariable in myosins are clearly distinguished: the N-terminal SH3 folding domain (residues 37–75) and the two surface loops, known as loop 1 (residues 201–215) and loop 2 (residues 615–637). A fourth area of divergence is in the lever arm IQ domain that binds the light chains and is consistent with the different LCs that bind each parent MyHC. Apart from these hypervariable regions, the sequence divergence is found throughout the motor domain but with hot spots showing higher levels of variation. The variations in sequence do cluster when mapped on to the three-dimensional structure of the motor domain (see Fig. 6). We will consider in detail the potential correlation of sequence and functional changes under “Discussion” but outline broad observations here. As expected from the sequence identity numbers quoted in the introduction, the EO-MyHC is distinct from the other three fast isoforms, which are very closely related; these three are also distinct from the two cardiac isoforms. Of note is that, apart from loop 2, which is part of the actin-binding domain, there are few changes in the actin-binding site among the four fast muscle isoforms. This is in contrast to the α and β isoforms where we previously pointed out that a change in the actin affinity between α and β correlated with several residue changes at the actin-myosin interface (18). Another distinction between the fast muscle and the cardiac isoforms is in the converter domain (residues 719–781). The two cardiac MyHCs are identical in this region, whereas the three type II MyHCs are identical to each other, except for two conserved changes at residues 751 (A/G) and 754 (D/E). The three skeletal type II isoforms differ from the cardiac isoforms, and the EO is different from both groups. Finally, we previously identified a region of the upper 50 kDa that is different between the α and β isoforms and could be important for the communication between the nucleotide and actin-binding sites (18). This region is labeled in the alignment as the exon 7 region by analogy with *Drosophila* muscle myosins II because this area is alternately spliced in the fly to generate different myosin II isoforms from a single gene (27). This region shows variation among not only the four fast isoforms but also the cardiac and EO isoforms. These key areas and their role in modifying the biochemical kinetic and mechanical properties of the isoforms will be considered under “Discussion.”

## DISCUSSION

**FIGURE 5.**
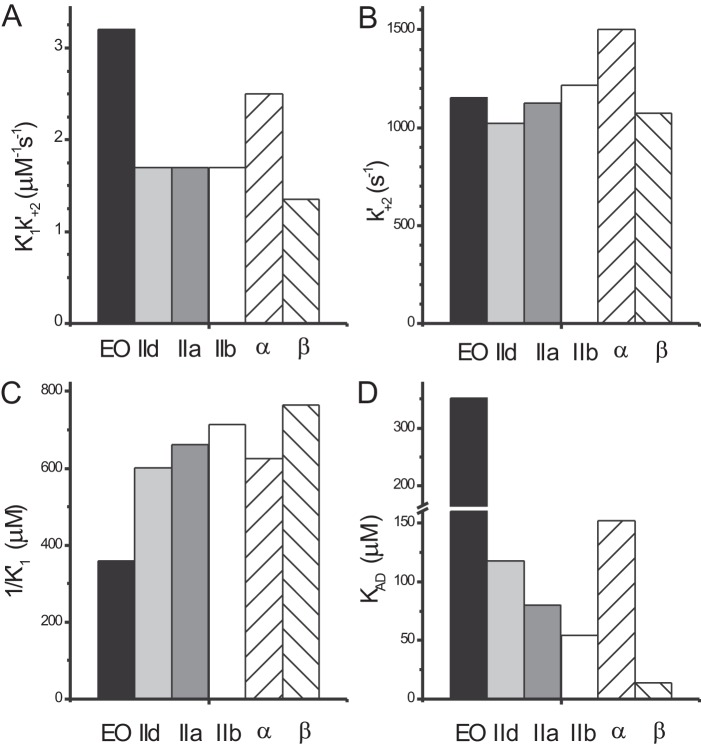
**Summary of kinetic data for human myosin isoforms IIa, IIb, IId and EO.** Comparison of rates and equilibrium constants for the human skeletal myosin S1 isoforms IIa, IIb, IId, and EO, together with the previously reported human α- and β-S1. *A*, the second order rate constant for ATP-induced dissociation of actin·S1 (*K*′_1_*k*′_+2_) is significantly faster for EO compared with the other skeletal isoforms. *B*, the maximum rate of ATP-induced dissociation of actin·S1 is very similar for all myosin isoforms. *C*, the apparent ATP affinity (1/*K*′*_1_*) is significantly different for EO compared with the other isoforms. *D*, in the presence of actin, the ADP affinity (*K_AD_*) is different for each human myosin isoform in the following order (weakest to strongest): EO > α > IId > IIa > IIb > β.

This study represents the first detailed kinetic measurements of homogeneous populations of single human skeletal MyHC isoforms IIa and IId and the first such study in any species of the specialized EO isoform. We also report the characterization of the human IIb isoform, which is not normally expressed in healthy human muscle. The data presented on the kinetics of single myosin isoforms are consistent with the distinct roles for individual isoforms in muscle contraction. The data are summarized in Table 1, and key parameters that change are shown in graphical form in Fig. 5.

All major muscle isoforms were isolated with a 1:1 ratio of mouse ELC and RLC attached. The MyHC-EO, however, had no LCs attached as reported by Resnicow *et al.* (11). The loss of LCs will abolish the velocity measured in a motility assay and can affect the stability of the protein (and hence apparent ATPase activity) but otherwise has only modest effects on the properties of the motor domain as shown by studies on both *Dictyostelium* myosin II and rabbit fast muscle myosin II motor domains (29, 30). We assume the MyHC-EO activity is normal, but this cannot be confirmed without a construct with LCs. Currently the appropriate LCs for this isoform are not defined.

### 

#### 

##### ATP-induced Detachment of S1 from Actin Is Similar for IIa, IIb, and IId but Much Faster for EO

The three fast skeletal muscle isoforms IIa, IIb, and IId are very similar with respect to the ATP-induced dissociation of actin·S1, because both steps (*steps 1* and *2* in Scheme 2) associated with ATP binding to actin·S1 are indistinguishable. This is significant because ATP-induced detachment has been suggested as a step that limits the maximum shortening velocity of a muscle fiber (15). Human MyHC-IId fibers contract at twice the speed of MyHC-IIa-containing fibers (14). MyHC-IIb is not expressed in human tissue, but other mammalian muscle fibers containing isoform IIb are reported to contract faster than fast skeletal fibers containing only MyHC-IIa or -IId (14). Because ATP-induced detachment appears invariant in the three human MyHC-S1 isoforms, this step is unlikely to limit the velocity of contraction in these muscle fibers. The previous step of ADP release could therefore be the event that limits cross-bridge detachment and therefore limits the velocity of muscle fiber contraction. Indeed, the ADP affinity for actin·S1 does vary (54–118 μm) and is weaker in the IId isoform (118 μm) than in the IIa isoform (80 μm), consistent with the differences in contraction speed. However, the ADP release measured here is a very fast step, and it has been argued previously that the step that limits velocity may be a second A·M·ADP complex, which is inaccessible to added ADP, because the equilibrium constant lies toward the weakly bound form of ADP (31, 32).

MyHC-EO is thought to be a very fast contracting isoform because of its presence in very fast twitch muscle fibers (7, 10). The data collected here are consistent with this view. The apparent second order rate constant for cross-bridge detachment, *K*′_1_*k*′_+2_, is double the value for the other three fast muscle isoforms, and ADP affinity for actin·S1 is weaker than the three fast muscle isoforms by almost 3-fold. This is consistent with very fast detachment from actin, and in this respect, EO myosin is similar to the indirect flight muscle myosin from *Drosophila* (*K_AD_* = 409 μm) (33). For fast muscle isoforms, like IIa/b/d, EO, and cardiac α, actin can rapidly displace ADP from S1, whereas in slow myosin isoforms like cardiac β, this process is less rapid. The thermodynamic coupling constant (*K_AD_*/*K_D_*) reflects this property, with fast myosins having values of *K_AD_*/*K_D_* ≥ 20, whereas slower isoforms typically have values of *K_AD_*/*K_D_* = 1–20 (34). For MyHC-EO, *K_AD_*/*K_D_* = 503, which is an extremely high value and indicative of very fast, efficient communication between the nucleotide and actin-binding sites. The other skeletal isoforms have more modest values with *K_AD_*/*K_D_* = 80 (IIa), 39 (IId), and 32 (IIb). The communication between the nucleotide-binding site and the actin-binding site goes through the 50-kDa domain, and subtle residue changes between various isoforms may contribute to different coupling constants between the two sites. We will consider the relationship between the sequences of the upper 50-kDa domain of the isoforms and the nucleotide binding properties below.

##### ATP Hydrolysis Is Similar for EO, IIa, IIb, and IId

The events monitored in the absence of actin for the four isoforms show a small variation in the apparent rate constant of ATP binding and the *k*_max_ for the fluorescence change on ATP binding (Table 1). Tryptophan fluorescence transients resulting from ATP binding to S1 have the potential to report multiple reactions, and for many myosins the signal can report both ATP binding governed by *K*_1_*k*_+2_ (Scheme 1, *steps 1* and *2*) and hydrolysis, *k*_+3_ +*k*_−3_ (Scheme 1, *step 3*). A characteristic of tryptophan fluorescence that reports both steps is that the observed amplitudes of the transients are smaller at high ATP concentrations as the ATP binding reaction becomes very fast and is lost in the dead time of the system. The observed amplitudes of the transient for fast skeletal muscle isoforms do fall by ∼50% between 20 and 400 μm ATP (Fig. 3*C*), consistent with the signal reporting both ATP binding and the hydrolysis step and *k*_max_ being defined by the rate of the hydrolysis step (*k*_max_ = *k*_+3_ + *k*_−3_). In contrast, the amplitudes observed for the EO-S1 reduced by a much smaller 10%. This is a surprise because there is no change in the number or location of the tryptophan residues in any of the MyHCs (supplemental Fig. S1). The expectation is therefore that the fluorescence changes should be similar for EO. The smaller loss of amplitude may suggest a change in the local environment of the tryptophan or that the equilibrium constant of the hydrolysis step is reduced compared with the other MyHCs. The ATP hydrolysis step is closely coupled to the recovery stroke of myosin and limits how long after cross-bridge detachment the myosin motor is again ready to interact with actin (35, 36). The values measured here would set this time as 5–7 ms (1/*k*_max_). The ADP affinity for S1 and the ADP release from S1 are not part of the normal cross-bridge cycle but may indicate how the nucleotide-binding pocket is altered in different isoforms and also gives information on the thermodynamic coupling constant (*K_AD_*/*K_D_*). The differences in ADP affinity are ∼3-fold with IId the weakest (3 μm) and EO the tightest (0.7 μm).

##### Human MyHC-IIb Has Some of the Kinetic Attributes of a Slower Myosin

Fast skeletal muscle myosins are largely characterized by rapid cross-bridge detachment kinetics. MyHC-IIb in rodents is associated with the fastest fiber types and is therefore thought to be very fast by these kinetic descriptors (1). Our measurements indicate that, surprisingly, human IIb binds ATP on the weaker end of the range for the fast skeletal isoforms and has significantly tighter ADP binding (*K_AD_*) than IIa or IId (*p* < 0.05). For both of these constants, the data would predict that IIb is the slowest of the three fast skeletal muscle isoforms. This is consistent with our earlier *in vitro* motility data for the human MyHC isoforms (11). It is conceivable that in humans the absence of MyHC-IIb expression has allowed genetic drift to a degenerate form. However, it remains a functional motor, and an alignment of human IIb with IIb sequences from rodents does not show any greater differences than a similar comparison of IIa or IId sequences.

##### Homology Models Predict Structural Differences between IIa, IIb, IId, and EO

The summary of the parameters measured for actin·S1 (Fig. 5) shows that there is a trend in the values of *K_AD_* from EO - IId - IIa - IIb from weak to tighter ADP affinity for the myosin isoforms. These values follow the trend for the shortening velocities measured for muscle fibers and for *in vitro* motility data from the same isoforms (11, 14). MyHC-β (skeletal muscle isoform I) has even tighter ADP affinity and slow velocities, whereas MyHC-α is similar to IId in nucleotide affinity. Sequence alignments (supplemental Fig. S2) point to some areas of structure that may be important in tuning function. Alignments of IIa, IIb, IId, and EO sequences show no changes in residues that are in direct contact with nucleotide or Mg^2+^, and therefore the different nucleotide-binding properties of the three isoforms for S1 or actin·S1 are not simply explained by changes in the nucleotide pocket itself. Loop 1 is a flexible region joining two α-helices that connect with two nucleotide-binding regions (P-loop and switch-1). The mobility of loop 1 has been correlated with the rate of ADP release with more flexible loops showing a weaker ADP affinity in myosin (37). Loop 1 is identical for the three fast muscle isoforms IIa, IIb, and IId except for two central residues 210 (I/P/V for the three isoforms, respectively), and 211 (T/A/T). Loop 1 of EO is one residue shorter and has three residue changes compared with IIa, IIb, and IId. Homology models predict a more ordered structure for loop 1 of IIa, IIb, and IId compared with EO in the post-power stroke conformation (supplemental Fig. S3), and this difference could play a role in the rapid ADP release for MyHC-EO. These loop 1 sequence differences are even more pronounced in comparison to the α and β isoforms.

**FIGURE 6.**
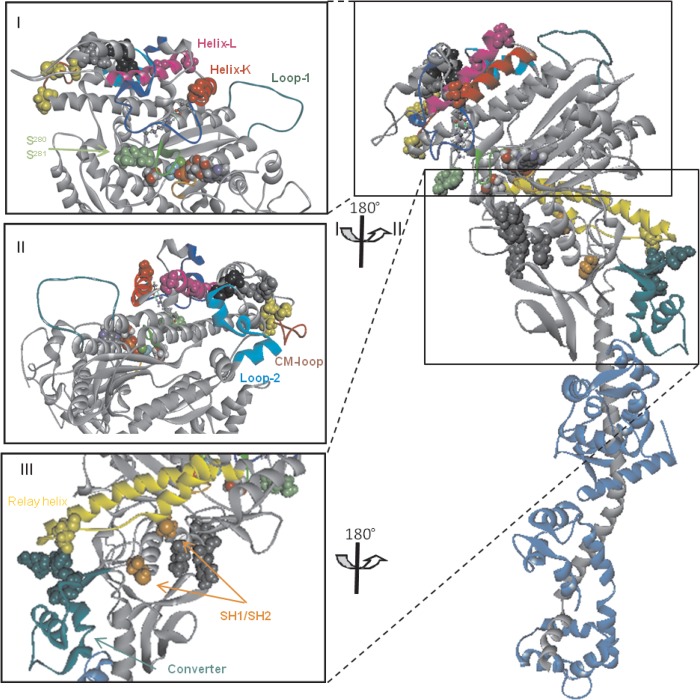
**Location of variable residues in the main motor domain of EO, compared with IIa/b/d.** EO is based on the scallop crystal structure (Protein Data Bank code 1kk8) with myosin heavy chain (*gray*), myosin light chains (*blue*), the bound nucleotide (space-filling model), P-loop (*orange*), and SW-1 (*light green*). *Panels I* and *II* (upper 50-kDa domain): switch-1 relay area residues Ser-280 and Ser-281 (*green*), SW-1 relay residue Arg-283 (ball and stick) makes a salt bridge toward Glu-327 located at the N terminus of helix K (*red*) and next to variable residue Ser-328 (*red*). Other variable residues are Asn-336 in helix K and Gly-350 in helix L (*pink*). Near the actin-binding site, a cluster of variable amino acids is located after helix L and next to the cardiomyopathy loop (*CM-loop*). These amino acids are between Gly-387 and Asn-418 for EO (Ala-388 to Thr-419 for IIa/b/d) and could perturb the CM loop interaction with actin and/or alter the signal communication between nucleotide-binding pocket and the actin-binding site. *Yellow residues*, located on the CM loop, can bind directly to actin (Gly-400, Cys-403, and Asn-418), whereas other variable residues, located further away from the CM-loop, can alter the communication between actin binding and nucleotide binding (Gly-387, Met-390, and Gly-391 in *black* and Glu-396 and Met-397 in *gray*). *Panel III*, converter-relay helix interface. Variable EO residues Phe-760, Arg-761, and Asn-764 are found at the interface of the converter region (*green*) and the relay loop (*yellow*) These residues are Tyr-760, Lys-761, and His-764 in IIa/b/d. The relay loop of EO and IIa/b/d shows only one change in the interface Glu-511 (*yellow*), which is Thr-512 for IIa/d. Variable residues close to the reactive thiols SH1 and SH2 (Cys-699 and Cys-709 for EO shown in *orange*) are depicted in *dark gray*: Val-687 (Ala), Asp-689 (Glu), Tyr-691 (Glu), and Met-694 (Leu). The sulfhydryl helix is thought to transduce the chemical energy provided by ATP hydrolysis into movement, and therefore changes in this region can alter the transduction signal.

An extensive area of the upper 50-kDa domain between switch-1 and the cardiomyopathy loop shows changes in sequence for the six isoforms. This is illustrated in Fig. 6 (*panels I* and *II*), where the position of the alternate residues are mapped onto a three-dimensional crystal structure of the myosin motor (scallop). The upper 50-kDa domain links the nucleotide-binding pocket with the actin-binding site (“upper jaw”), and changes in actin or nucleotide binding at the two sites are communicated through this domain. We previously identified residue changes between α and β cardiac myosin sequences in this area (residues 297–327), and these cardiac isoforms have markedly different affinities for ADP in the presence of actin (20 and 120 μm) and differences in the coupling of ADP and actin binding (*K_AD_*/*K_D_*) (18). This is also the area that is alternately spliced in *Drosophila* (exon 7; see supplemental Fig. S1) to generate myosin with differing properties including the affinity of actin·myosin for ADP (38). The area of sequence change is slightly larger across the six human isoforms shown here and includes regions just before and just after the marked exon 7 region. We will point out some areas that may be of special interest.

One such variable residue is Ser-328 in MyHC-EO which is a known cardiomyopathy site in β-myosin (Ala-326 in β) (39). Ser-328 is located at the N terminus of helix K (Fig. 6, *panel I*), close to the highly conserved residues Asp-327 (that forms a salt bridge with Arg-283) and Glu-330. Both corresponding residues for Arg-283 and Glu-330 in β-myosin (Arg-281 and Glu-328) are also known cardiomyopathy sites, emphasizing their functional importance (40, 41). Arg-283 and its neighboring residues are thought to relay changes in the nucleotide-binding pocket, switch in particular, toward the actin-binding site through the upper 50-kDa domain (27). Two residues close to Arg-283 in this area vary between EO (Ser-280 and Ser-281) and the three skeletal myosins II a/b/d (Lys-281 and Ala-282). The homology models predict that the two serine residues in EO increase the number of interactions between the relay area and switch-1, which may allow more efficient communication between the nucleotide-binding pocket and the actin-binding site (highlighted in *green* in Fig. 6, *panel I*). It is worth noting that the very fast IFI myosin from *Drosophila* shares the serine residue at position 280 in common with EO.

The exon 7 area ends in the middle of helix K, and a short loop joins this to helix L (Fig. 6, *panel I*). We have argued previously (27) that this exon 7 region and its links to helix L are important in coupling the nucleotide-binding pocket and the actin-binding sites, and therefore the same argument can be made for the role of these sequence changes in skeletal myosin. Helix L contains one variable residue, Gly-350 for EO (Fig. 6, *panel II*, *pink*). A similar variable cluster of three residues was found in Helix L for the two cardiac myosin isoforms, residues 347–349 (NSM in β and AGV in α) (18), and lies between two highly conserved lysines at 346 and 351 of which Lys-351 in MyHC-β also is a human cardiomyopathy site. Another interesting cluster of variable amino acids is located after helix L and next to the cardiomyopathy loop (Fig. 6, *panels I* and *II*, *yellow* and *gray*). These are between Gly-387 and Asn-418 for EO (Ala-388 to Thr-419 for IIa/b/d), and again these changes could perturb the cardiomyopathy loop interaction with actin and/or alter the signal communication between the nucleotide-binding pocket and the actin-binding site.

The converter area is identical for the three fast skeletal muscle isoforms but does show variations compared with MyHC-EO. The converter is important in converting events near the nucleotide pocket into a swing of the lever arm; that is, in amplifying events at the nucleotide pocket into large scale movement. Of particular interest are the converter residues located at the interface between the converter and the relay loop (Fig. 6, *panel III*, *green*). The relay loop of EO and IIa/b/d shows only one variable residue in the interface Glu-511 (Fig. 6, *panel II*, *yellow*). The homology models show more contacts between the converter and the relay domain for EO compared with the other three isoforms. This indicates a stronger link between the nucleotide-binding pocket and the converter region, and this could alter the mechanical coupling of the force generation. Located near the converter domain are a few variable residues (Fig. 6, *panel III*, *dark gray*) close to the reactive thiols SH1 and SH2 (Fig. 6, *panel III*, *orange*). The sulfhydryl helix is thought to transduce the chemical energy provided by ATP hydrolysis into movement, and therefore changes in this region can alter the transduction signal.

In summary, this study describes for the first time the transient kinetics of adult human fast skeletal sarcomeric myosins. Human EO is an extremely fast myosin with more than 3-fold weaker ADP affinity compared with three other human fast skeletal isoforms (IIa, IId, and IIb). Isoform IIb is unusually slow compared with rodent isoforms. Sequence alignments and homology modeling identify a key region in the upper 50-kDa domain important in tuning the signaling between the nucleotide pocket and the actin-binding site for the different isoforms.

## Supplementary Material

Supplemental Data

## Footnotes

6 The abbreviations used are:

MyHC: myosin heavy chain
LC: light chain
ELC: essential light chain
RLC: regulatory light chain
S1: myosin subfragment 1
EO: extra-ocular
eGFP: enhanced green fluorescent protein.
