# Supplementary material for: The Superfast Human Extraocular Myosin Is Kinetically Distinct from the Fast Skeletal IIa, IIb, and IId Isoforms
Source: J Biol Chem. 2013 Aug 1;288(38):27469–79. doi: 10.1074/jbc.M113.488130 (PMC3779741; doi:10.1074/jbc.M113.488130)
Supplement: Supplemental Data [file supp_288_38_27469__index.html]

The superfast human extra-ocular myosin is kinetically distinct from the fast skeletal IIa, IIb and IId isoforms — The Superfast Human Extraocular Myosin Is Kinetically Distinct from the Fast Skeletal IIa, IIb, and IId Isoforms — Kinetics of Human Sarcomeric Myosins — Supplemental Data 

# The Superfast Human Extraocular Myosin Is Kinetically Distinct from the Fast Skeletal IIa, IIb, and IId Isoforms

## Supplemental Data

**Files in this Data Supplement:**

- Supplemental data pdf (.pdf, 692 KB) - This is the pdf that contains all (revised) supl data that is to be published with the article.
