# Supplementary material for: The Superfast Human Extraocular Myosin Is Kinetically Distinct from the Fast Skeletal IIa, IIb, and IId Isoforms
Source: J Biol Chem. 2013 Aug 1;288(38):27469–79. doi: 10.1074/jbc.M113.488130 (PMC3779741; doi:10.1074/jbc.M113.488130)
Supplement: Supplemental Data [file supp_M113.488130_jbc.M113.488130-1.pdf]

Supplementary Material

The superfast human extra-ocular myosin is kinetically distinct from the fast skeletal IIa, IIb and IId isoforms.

Marieke J. Bloemink[1#], John C. Deacon[2, 3#], Daniel I. Resnicow[2,4], Leslie A. Leinwand[2] and Michael A. Geeves[1].

Developmental Biology, University of Colorado, Boulder, CO, USA,[3]Current address: Dept of Molecular Biophysics and Biochemistry, Yale University, New Haven; CT, USA.[4]Current address: X-Chem, Inc., 100 Beaver Street Suite 101, Waltham, MA 02453

[#] Contributed equally to this work.

†Corresponding Authors' Email: m.a.geeves@kent.ac.uk; leslie.leinwand@colorado.edu

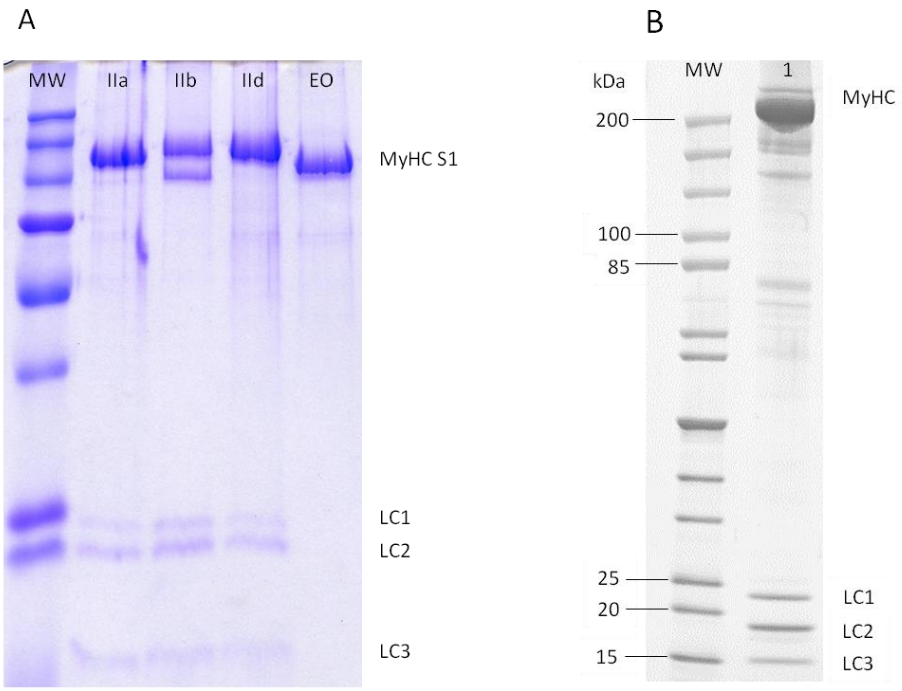

Figure S1: Expression and purification of the human skeletal myosin II isoforms. (A) Example of a typical Coomassie Blue stained SDS-PAGE gel with MyHC-S1 fractions corresponding to the human skeletal isoforms MyHC-IIa-eGFP-6xHis (IIa), MyHC-IIb-eGFP-6xHis (IIb), MyHC-IId-eGFP-6xHis (IId) and MyHC-EO-eGFP-6xHis (EO) together with a molecular weight marker (MW). The human skeletal MyHC S1 IIa, IIb and IId co-purify with the host mouse LCs, whereas no light chains are found associated with the human MyHC-EO, as reported previously (Resnicow, et al PNAS *107*, pp1053-1058, 2010). Note that in some preparations the MyHC appears as a double band or has a contaminant of similar size to the HC, as shown in this example for MyHC-IIb. The behaviour of the protein in the transient experiments was similar irrespective of the presence of the doublet. (B) A typical gel of purified rabbit myosin (lane 1), prepared from tissue (mixed isoforms), is shown with its associated light chains. The ratios between similar sizes LCs in the skeletal myosin isoforms are similar to those seen in rabbit myosin. This is compatible with the presence of both essential (LC1 & LC3) and regulatory light chains in a ratio of close to 1:1 when allowing for differences in MW.

Table S1: Ratio of light chains (LC) and Heavy Chains (HC) LC/HC in motor domain preparations. LC1, LC2 and LC3 represent Essential Alkali-1 Light Chain, Regulatory Light Chain, Essential Alkali-2 Light chain respectively. Densities were measured using the gels shown in Figure S1 (A/B).

	human MyHC IIa	human MyHC IIb	human MyHC IId	human MyHC EO	Rabbit myosin
HC	1.0	1.0	1.0	1.0	1.0
LC1<br>~25 kDa	0.060	0.069	0.050	-	0.104
LC2<br>~20 kDa	0.131	0.165	0.124	-	0.158
LC3<br>~18 kDa	0.126	0.096	0.086	-	0.092
Total LC	0.317	0.330	0.260		0.354
LC2/total LC	0.42	0.50	0.47		0.45

Table S1 shows the ratio of densities of mouse myosin light chains to human heavy chain bands on SDS-PAGE stained with Coomassie blue (see also Figure S1A). The values are approximate because there is a wide range of molecular weights (MW) on the gel and loading and staining is not always equivalent. As reported previously (Resnicow et al 2010) the human skeletal MyHC motor domain purifies along with the endogenous mouse LCs and these were identified by mass spectroscopy as MLC1F, MLC2F, MLC3F, and MLC1A. These are also known as the Essential Alkali-1 Light Chain, Regulatory Light Chain, Essential Alkali-2 Light chain, Atrial/Fetal Light Chain respectively. As reported by Resnicow et al 2010, no C_2C_{12} light chains are found associated with the human MyHC-EO.

The results for a typical gel of purified rabbit myosin, prepared from tissue (mixed isoforms), is shown in Table S1 and Figure S1B.The ratios between similar size LCs are similar to those seen in rabbit myosin. This is compatible with the presence of both essential (LC1 & LC3) and regulatory light chains in a ratio of close to 1:1 when allowing for differences in MW. Note that in some preparations the MyHC appears as a doublet or has a contaminant of similar size to the HC, as shown in this example for MyHC-IIb. The behaviour of the protein in the transient experiments was similar irrespective of the presence of the doublet. Different preparations gave apparent variations in the ratios of the different LC/HC but again there were no observable differences in kinetic behaviour that correlated with the change in LC content.

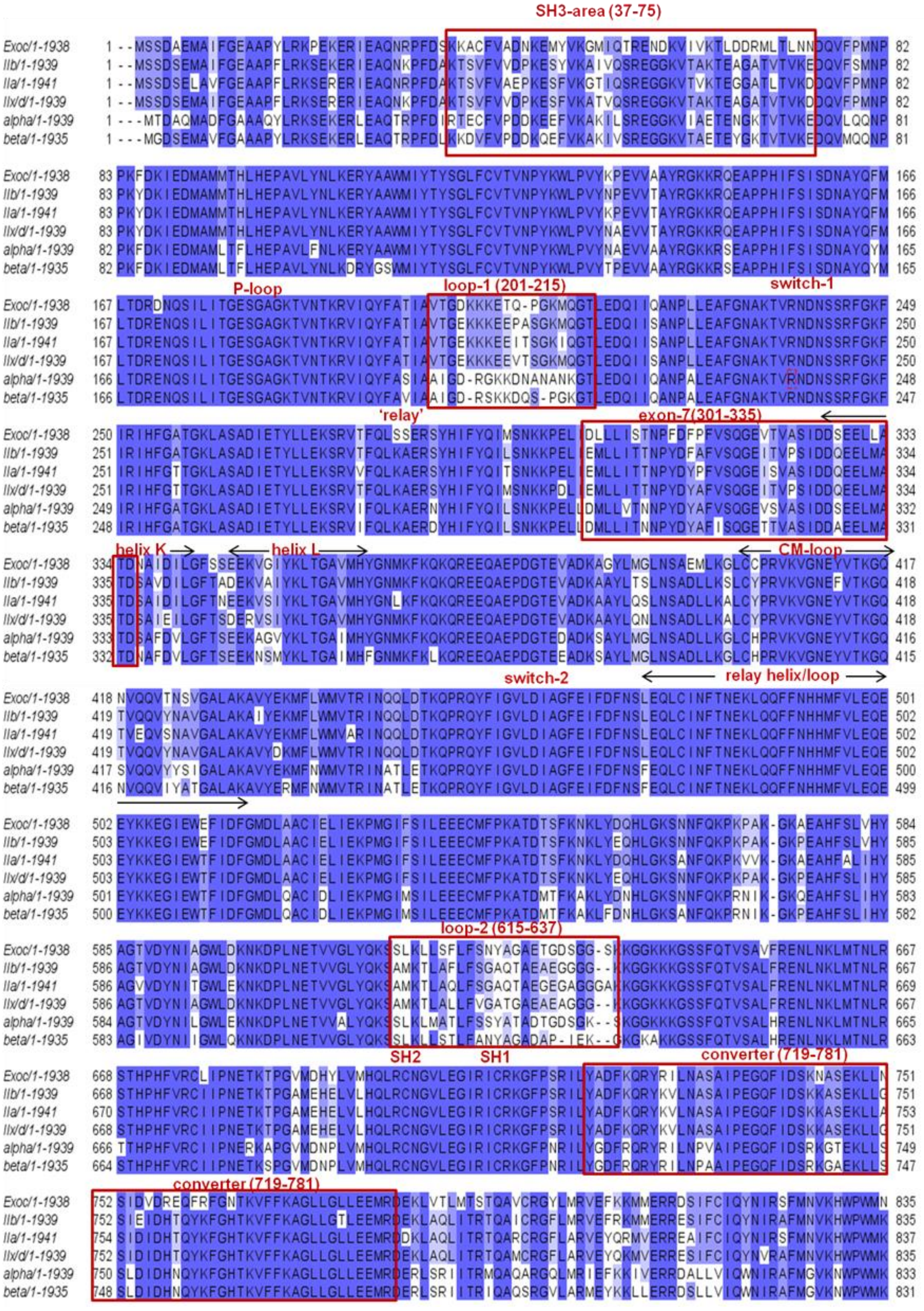

Figure S2: Alignment of human skeletal myosin isoforms IIa, IIb, IId and EO, together with the two human cardiac isoforms, α and β. Coloring represents residue conservation with conserved residues in dark blue, semi-conserved residues in light-blue and non-conserved residues in white.

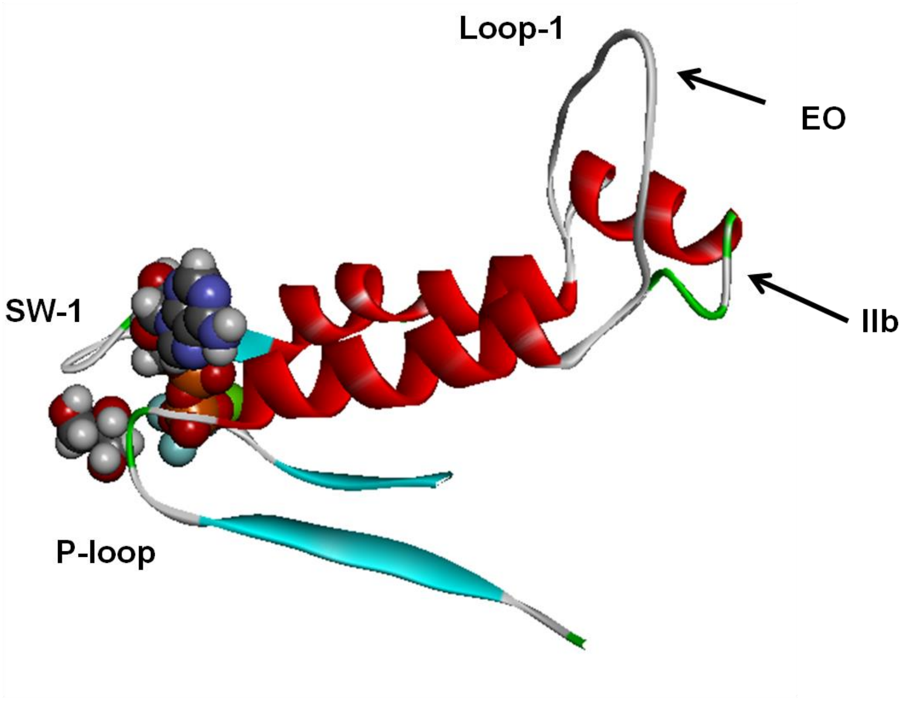

Figure S3: Homology models predict structural differences between the human isoforms EO, IIa, IIb and IId in Loop-1. Overlay of the loop-1 area of homology models of extraocular S1 (EO) and S1-IIb (built using post-powerstroke scallop myosin (PDB code 1kk8) as template) showing a helical structure of loop 1 for IIb, which is not seen in EO. The bound nucleotide is shown as *solid spheres*, and switch-1 (*SW-1*) and the phosphate binding loop (*P-loop*) are also indicated. The altered structure of loop-1 may affect its flexibility and ultimately affect the ADP release rate with faster release for EO (less ordered loop-1 structure) and slower release for IIb (more rigid loop-1 structure). Homology models for IIa and IId show similar loop-1 structures as seen for IIb.
